# Association of supine apical vertebral rotation with three-dimensional deformity and global spinal alignment in adult degenerative scoliosis: a retrospective study

**DOI:** 10.3389/fmed.2026.1919114

**Published:** 2026-08-19

**Authors:** Xueneng Yang, Ruijuan Li, Junfei Liu, Bo Li, Jun Shu

**Affiliations:** 1 Xinjiang Medical University First Affiliated Hospital, Xinjiang Medical University, Urumqi, Xinjiang, China; 2 Department of Traumatology, Second Affiliated Hospital of Kunming Medical University, Kunming, Yunnan, China; 3 Hanche Medical Aesthetic Hospital, Kunming, Yunnan, China; 4 Department of Geriatric Orthopedics, Affiliated Hospital of Yunnan University, Kunming, Yunnan, China

**Keywords:** adult degenerative scoliosis, apical vertebral rotation, global imbalance, spinal alignment, three-dimensional deformity

## Abstract

**Objective:**

To evaluate the clinical and radiographic significance of supine CT-measured apical vertebral rotation (AVR) in adult degenerative scoliosis (ADS).

**Methods:**

A total of 163 patients with ADS were retrospectively included. Clinical outcomes included low back pain visual analog scale (VAS), leg pain VAS, Japanese Orthopedic Association (JOA) score, and Oswestry Disability Index (ODI). Radiographic parameters included sagittal vertical axis (SVA), coronal vertical axis (CVA), apical vertebral translation (AVT), lumbar lordosis (LL), thoracolumbar kyphosis, pelvic parameters, coronal Cobb angle, and AVR. AVR was measured on supine axial CT images using Ho's method. Reproducibility was assessed using ICC(2,1). Correlation, multivariable regression, sensitivity, and exploratory interaction analyses were performed.

**Results:**

All radiographic measurements showed excellent reproducibility, with an AVR ICC(2,1) of 0.999. AVR correlated with worse pain and functional scores and with multiple radiographic parameters In multivariable linear regression, SVA, AVT, and Cobb angle were independently associated with AVR. When SVA and CVA were analyzed as continuous outcomes, AVR remained independently associated with SVA, but not with CVA. In the threshold-defined global imbalance analysis, AVR remained independently associated with global imbalance after excluding SVA and CVA from the multivariable model (OR = 1.108, 95% CI: 1.036–1.184, *P* = 0.003). Sensitivity analyses supported the robustness of this association. No significant interaction was found between AVR and SVA or CVA.

**Conclusions:**

Supine CT-measured AVR is associated with clinical symptoms and three-dimensional deformity parameters in ADS. It provides additional axial information beyond coronal and sagittal assessment, but should not be used alone to explain global imbalance or predict deformity progression. Because AVR was measured on supine CT whereas SVA/CVA were obtained from standing radiographs, and some clinical confounders were unavailable for adjustment, these findings should be interpreted as associations rather than causal evidence.

## Graphical Abstract

**Figure:**
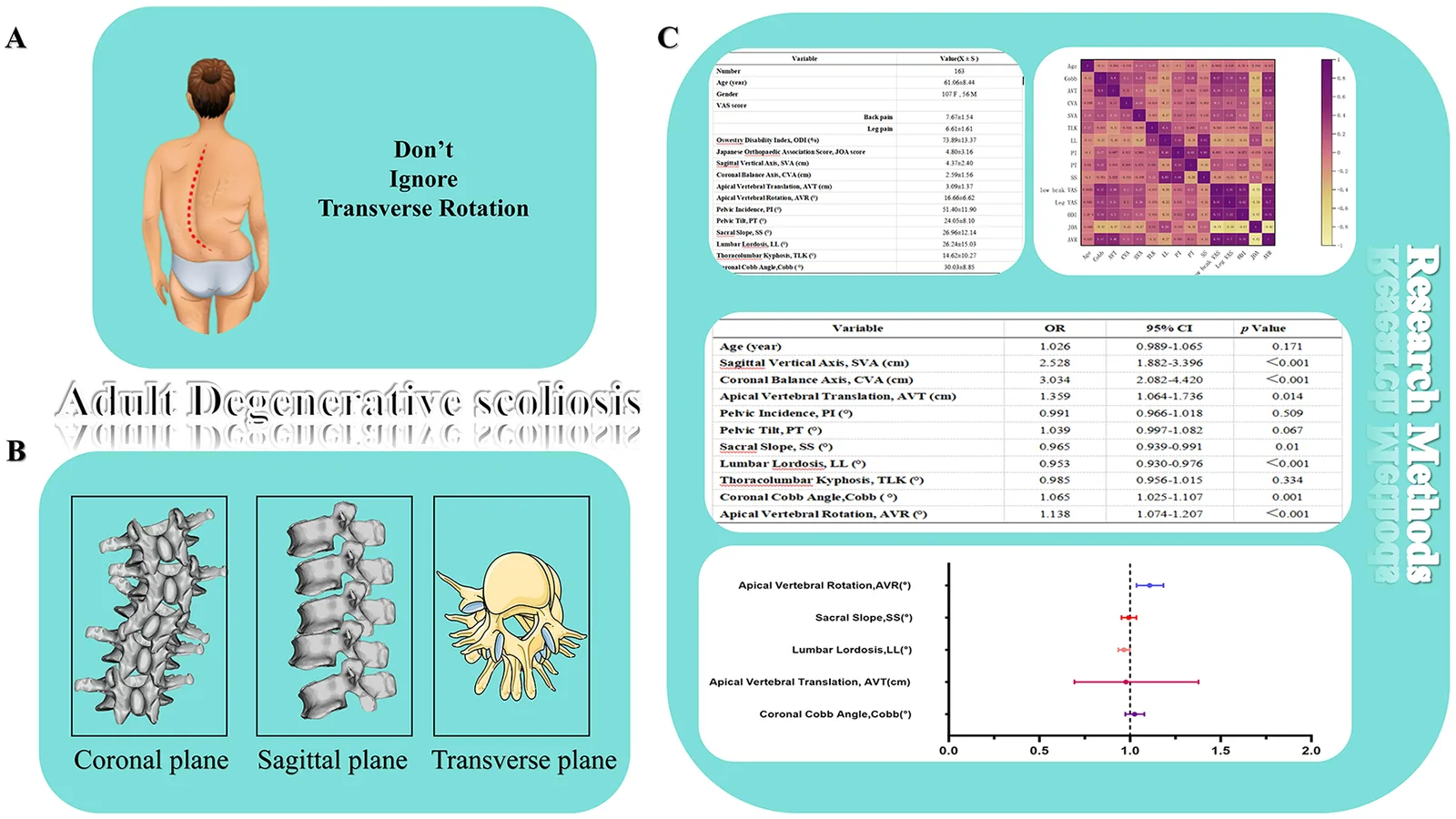
Apical certebral rotation in adult degenerative scoliosis: a predictive study. A. Schematic diagram of the study population, illustrating the role of vertebral rotation in adult degenerative scoliosis. B. Illustration of the three-dimensional deformity in adult degenerative scoliosis. C. Research methods used for vertebral rotation analysis: correlation analysis and logistic regression.

## 1 Introduction

Adult degenerative scoliosis (ADS) is a complex three-dimensional spinal deformity caused by degenerative changes in the spine and surrounding structures (1, 2). It affects 6%−68% of individuals over the age of 60 (3). The main cause is asymmetric disc degeneration, which leads to coronal and sagittal imbalance. In addition, subluxation of the facet joints results in vertebral rotation and lateral translation in the horizontal plane (4, 5). These spatial deformities interact and compromise spinal stability, contributing to spinal canal stenosis, nerve root compression, postural imbalance, and gait disturbance. These changes affect patients' daily function and quality of life (6). ADS has become a growing public health concern in aging populations. In 2010, the cost of ADS-related treatment in the United States reached $958 million, with a continuous upward trend in recent years (7). Therefore, establishing a reliable evaluation system that accurately quantifies three-dimensional deformity and supports clinical decision-making is essential for improving outcomes and optimizing healthcare resource allocation.

In current clinical practice for ADS, sagittal vertical axis (SVA) and coronal vertical axis (CVA) are established as key parameters for assessing global imbalance and guiding surgical decisions (8–10). However, the clinical value of apical vertebral rotation (AVR), an important component of the three-dimensional deformity in ADS, remains unclear. Previous studies have shown that AVR is closely associated with facet joint degeneration and neural foraminal stenosis (5). It may also contribute to pain and functional impairment (11). However, the relationships between AVR, global spinal alignment, clinical symptoms, and three-dimensional deformity characteristics in ADS have not been fully evaluated (12). Therefore, this study used spinal CT images to quantify supine CT-measured AVR with Ho's method. Clinical symptom scores and standing full-spine radiographic parameters were also collected. Correlation analysis and multivariable regression analyses were performed to evaluate the relationships between AVR, clinical symptoms, three-dimensional deformity parameters, and global spinal alignment. This study aimed to clarify the radiographic and clinical significance of AVR in the three-dimensional assessment of ADS. The findings may help improve comprehensive evaluation of ADS and provide reference information for individualized treatment decision-making and surgical planning.

## 2 Materials and methods

### 2.1 Study period and location

Data collection was conducted from July 2022 to November 2025 at the Second Affiliated Hospital of Kunming Medical University.

### 2.2 Inclusion and exclusion criteria

Inclusion criteria: (1) Availability of full-spine coronal and sagittal radiographs, as well as axial CT scans suitable for parameter measurement; (2) Coronal Cobb angle greater than 10°; and (3) Skeletal maturity.

Exclusion criteria: (1) Spinal deformities not caused by degeneration, such as idiopathic scoliosis, post-traumatic deformity, ankylosing spondylitis, or tumor-related deformity; (2) History of surgery involving the spine, hip, or pelvis.

### 2.3 Study population, outcome definition, and grouping

**Figure 1:**
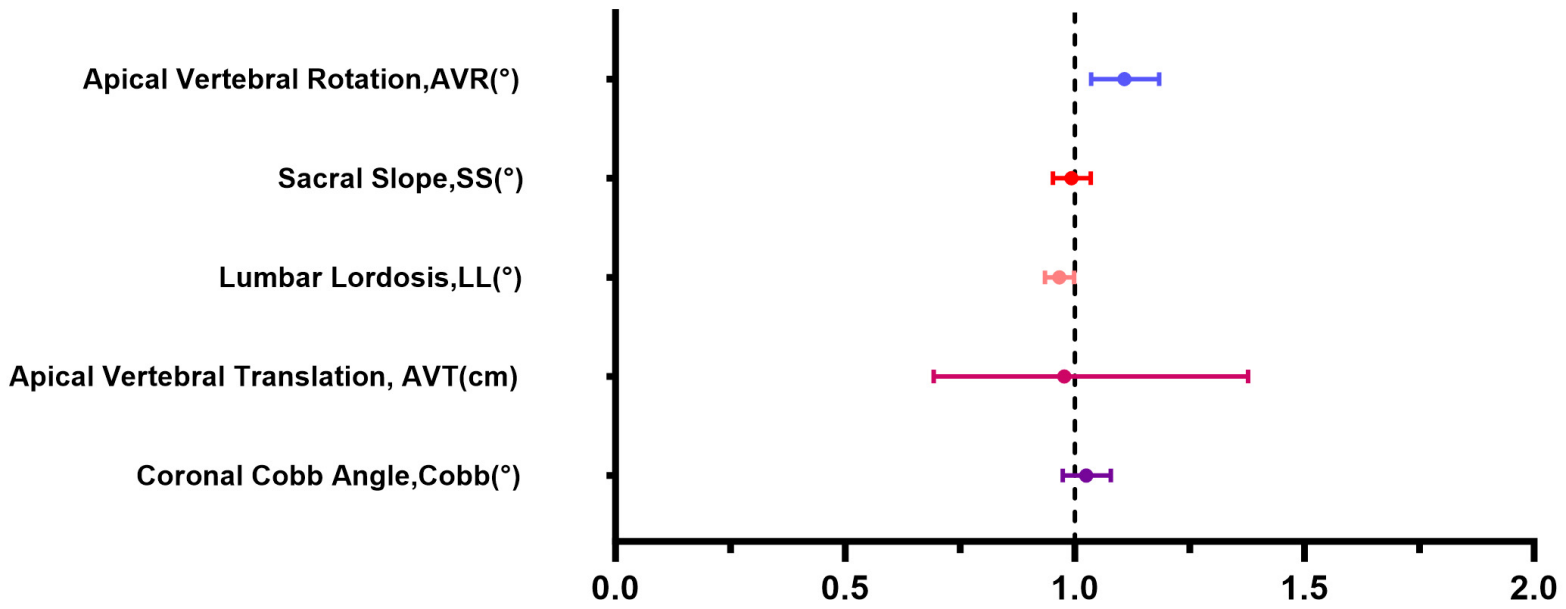
Flow diagram of patient selection and analysis cohort. ADS, adult degenerative scoliosis; CT, computed tomography; CVA, coronal vertical axis; SVA, sagittal vertical axis.

A total of 212 patients diagnosed with ADS at the Second Affiliated Hospital of Kunming Medical University between July 2022 and November 2025 were reviewed. After applying the inclusion and exclusion criteria, 49 patients were excluded, including 19 patients whose CT scans were performed at outside hospitals, 22 patients with a history of spine, hip, or pelvic surgery, and 8 patients who had trauma-related spinal deformity despite a diagnosis of ADS. Finally, 163 patients with ADS were included in the study. To evaluate the association between vertebral rotation and global spinal imbalance, CVA ≥ 3 cm and/or SVA ≥ 5 cm were used as the criteria for imbalance, based on previous studies and clinical guidelines. Accordingly, patients were divided into two groups: the imbalance group (*n* = 91, CVA ≥ 3 cm and/or SVA ≥ 5 cm) and the balance group (*n* = 72, CVA < 3 cm and SVA < 5 cm), which served as the basis for further statistical analysis (9, 13).This study was conducted in accordance with the Declaration of Helsinki (Figure 1).

### 2.4 Imaging evaluation

All radiographic images were reviewed and measured by consensus by two senior spine surgeons and one senior radiologist. All radiographic parameters were determined using a consensus-based measurement approach. Specifically, the three experts jointly discussed the measurement level, anatomical landmarks, and measurement method, and the final values were recorded after agreement was reached. Sagittal and coronal parameters were measured on full-length standing spinal radiographs, whereas axial parameters were measured on spinal CT images. All included patients were undergoing preoperative evaluation for surgical treatment because of symptomatic and structurally complex adult degenerative scoliosis. Spinal CT was clinically indicated as part of the routine preoperative assessment to characterize the three-dimensional osseous morphology of the spine, vertebral rotation, degenerative changes, and anatomical features relevant to surgical planning. The present study retrospectively analyzed these clinically acquired CT images, and no additional CT examination was performed solely for AVR measurement or research participation [Lumbar CT scans were performed using a 64-row multidetector spiral CT scanner. Patients were positioned supine with a soft cushion placed beneath both knees to relax the lumbar spine and maintain a comfortable physiological curvature. The scout line was aligned at the level of the iliac crest, and the scan range extended from the superior endplate of L1 to the inferior endplate of S2. The scanning parameters were as follows: tube voltage, 120 kV; automatic tube current modulation with a noise index of 11; tube current range, 50–300 mA; pitch, 0.938; detector collimation, 0.625 mm; field of view, 240 mm; and matrix, 512 × 512. After scanning, two sets of axial images were reconstructed. Soft-tissue images were reconstructed using a standard soft-tissue algorithm with a slice thickness of 2.5 mm and an interval of 2.5 mm. Bone images were reconstructed using a high-resolution bone algorithm with a slice thickness of 1.0 mm and an overlapping interval of 0.5 mm. Standard window settings were as follows: soft-tissue window, window width 350 HU and window level 50 HU; bone window, window width 2,000 HU and window level 400 HU. Sagittal and coronal multiplanar reconstruction (MPR) images were simultaneously generated with a reconstruction thickness of 3 mm. No intravenous contrast agent was administered during the examination]. The measurement methods for each parameter are described below.

#### 2.4.1 Full-length standing anteroposterior and lateral spinal radiographs

**Figure 2:**
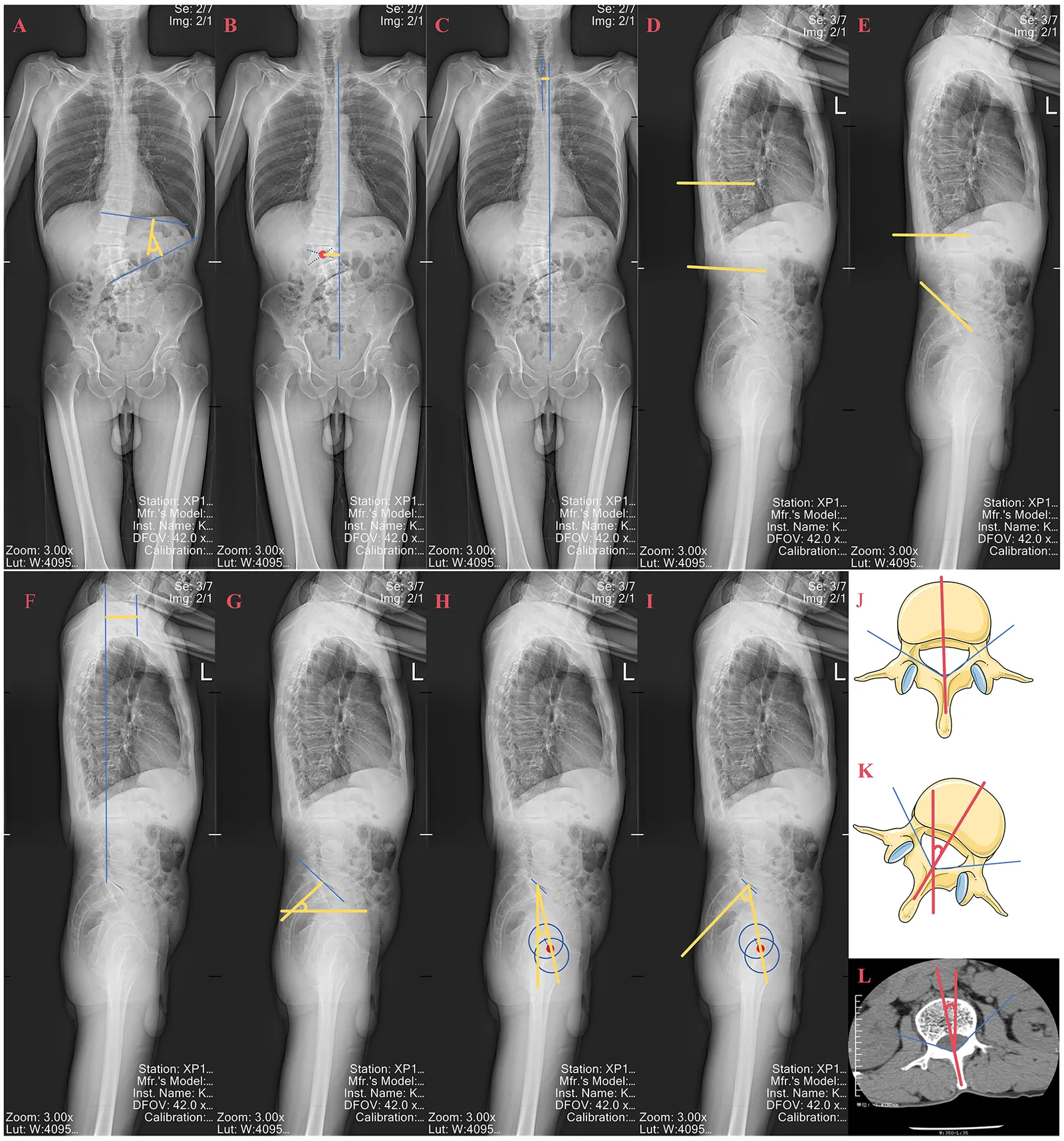
Measurement methods of study parameters. **A**: Coronal cobb angle. **B**: Apical vertebral translation (AVT). **C**: Coronal vertical axis (CVA). **D**: Thoracolumbar kyphosis (TLK). **E**: Lumbar lordosis (LL). **F**: Sagittal vertical axis (SVA). **G**: Sacral slope (SS). **H**: Pelvic tilt (PT). **I**: Pelvic incidence (PI). **J, K, L**: Apical vertebral rotation (AVR).

(1) Coronal parameters (see Figure 2):

① Coronal Cobb angle (Cobb): measured on full-length standing anteroposterior radiographs. Lines are drawn along the superior endplate of the most tilted upper vertebra and the inferior endplate of the most tilted lower vertebra. The angle between these two lines or their perpendiculars is recorded.
② Apical vertebral translation (AVT): the horizontal distance from the midpoint of the apical vertebra to the central sacral vertical line (CSVL) on the coronal radiograph.
③ CVA: the horizontal distance between the C7 plumb line and the CSVL on the coronal radiograph.

(2) Sagittal parameters:

① Thoracolumbar kyphosis (TLK): the sagittal angle between the superior endplate of T10 and the inferior endplate of L2.
② Lumbar lordosis (LL): the angle between the superior endplate of L1 and the superior endplate of S1 on full-length lateral radiographs.
③ SVA: the horizontal distance from the C7 plumb line to the posterosuperior corner of the S1 vertebral body.
④ Pelvic incidence (PI): the angle between the line connecting the midpoint of the S1 superior endplate and the center of the femoral heads, and the line perpendicular to the S1 superior endplate at its midpoint. If the femoral heads are not superimposed, the midpoint of the line connecting both femoral head centers is used.
⑤ Pelvic tilt (PT): the angle between the vertical line and the line connecting the midpoint of the S1 superior endplate to the center of the femoral heads. If the femoral heads are not superimposed, the midpoint between both femoral head centers is used.
⑥ Sacral slope (SS): the angle between the tangent to the S1 superior endplate and the horizontal line on lateral radiographs.

(3) Axial parameter

① AVR: measured on axial CT images using Ho's method. The angle is formed between the bisector of the angle created by two lines—one connecting the inner borders of the laminae and the other connecting the inner borders of the pedicles—and the sagittal plane. If the apical level is located at the intervertebral disc, the average rotation of the adjacent vertebrae is used to represent the AVR.

### 2.5 Statistical analysis

All data were analyzed using SPSS version 27.0. The normality of continuous variables was tested. Variables with a normal distribution were expressed as mean ± standard deviation (SD) and compared using the independent samples *t*-test. Variables not normally distributed were expressed as median and interquartile range (IQR), and compared using the Mann–Whitney U test. Categorical variables were presented as frequencies and compared using the chi-square test.

#### 2.5.1 Reproducibility analysis of radiographic measurements

To evaluate the reproducibility of consensus-based radiographic measurements, 30 patients were randomly selected for repeated measurement analysis. The same expert panel repeated the measurements of Cobb angle, AVT, CVA, SVA, TLK, LL, PI, PT, SS, and AVR using the same measurement protocol after an interval of more than 1 month. The intraclass correlation coefficient [ICC(2,1)] with 95% confidence intervals was used to assess the reproducibility of each radiographic parameter. ICC(2,1) was calculated using a two-way random-effects model, absolute agreement, and single-measurement values.

#### 2.5.2 Correlation analysis

Pearson correlation analysis was used to assess the relationships between AVR and clinical or radiographic parameters. If the variables were not normally distributed, Spearman correlation analysis was used instead. The correlation results were used to describe the initial associations between AVR and each parameter and to guide the subsequent regression models.

#### 2.5.3 Regression model construction

Because this was a retrospective study, BMI, bone mineral density, the degree of paraspinal muscle degeneration, and parameters related to lower-limb/pelvic compensation were incompletely recorded in the original medical records and imaging data; therefore, these variables could not be included in the multivariable adjusted models.

① *Linear regression analysis with AVR as the dependent variable*

To identify radiographic factors independently associated with apical vertebral rotation, AVR was treated as a continuous dependent variable in linear regression analysis. Univariable linear regression was performed first. Radiographic variables with *P* < 0.05 in the univariable analysis were then entered into a multivariable linear regression model. The enter method was used for the multivariable model. Results were reported as unstandardized regression coefficients (*B*), 95% confidence intervals (CIs), standardized regression coefficients (β), and *P* values. Multicollinearity was assessed using the variance inflation factor (VIF), and VIF < 5 was considered to indicate no marked multicollinearity.

② *Linear regression analysis with SVA and CVA as continuous dependent variables*

Because SVA and CVA are continuous radiographic parameters, linear regression analyses were performed with SVA and CVA as separate dependent variables. This was done to avoid information loss caused by dichotomizing these parameters only by clinical thresholds. Univariable linear regression was performed first. Variables with *P* < 0.05 in the univariable analysis were then entered into multivariable linear regression models. As the main variable of interest, AVR was used to assess its independent association with SVA or CVA. The enter method was used for the multivariable models. Results were reported as B, 95% CI, β, and *P* values.

③ *Logistic regression analysis of threshold-defined global imbalance*

Patients were classified into the global imbalance group and the non-imbalance group according to CVA ≥ 3 cm and/or SVA ≥ 5 cm. Global imbalance status was used as the binary dependent variable in logistic regression analysis. Univariable logistic regression was first used to assess the association between each variable and global imbalance status. Crude odds ratios (ORs) and 95% confidence intervals (CIs) were calculated. Variables with *P* < 0.05 in the univariable analysis were then entered into a multivariable logistic regression model using the enter method. Adjusted ORs and 95% CIs were reported.

④ *Sensitivity analysis*

To assess the robustness of the main findings and to reduce the potential influence of classification instability caused by cases near the clinical thresholds, sensitivity analyses were performed. First, sagittal imbalance was defined using SVA ≥ 5 cm alone, and sagittal imbalance status was used as the binary dependent variable to construct a multivariable logistic regression model in the full cohort. Second, to reduce the potential influence of patients with SVA or CVA values close to the clinical thresholds on the classification of global imbalance, patients with SVA between 4 and 6 cm or CVA between 2.5 and 3.5 cm were excluded, and the logistic regression analysis for threshold-defined global imbalance was repeated. As the main variable of interest, AVR was used to evaluate its association with sagittal imbalance and global imbalance in the sensitivity analyses. The enter method was used for the multivariable models, with Cobb angle, AVT, LL, SS, and AVR included in the models. Results were reported as ORs, 95% CIs, and *P* values.

⑤ *Interaction analysis*

To explore possible interactions between AVR and global alignment parameters, logistic regression models were built with interaction terms. Two interaction terms were tested separately: AVR × SVA and AVR × CVA. A *P* value < 0.05 for the interaction term was considered to indicate a statistically significant interaction. Because SVA and CVA were also used to define global imbalance status, this analysis was interpreted as exploratory.

All tests were two-sided, and a *P* value < 0.05 was considered statistically significant.

## 3 Results

### 3.1 Baseline characteristics

A total of 163 patients with ADS were included in this study, comprising 56 males and 107 females, with a mean age of 61.06 years (Table 1). According to the criteria of CVA ≥ 3 cm and/or SVA ≥ 5 cm, 72 patients were classified into the balanced group and 91 into the imbalanced group. Compared with the balanced group, the imbalanced group had significantly higher low back pain VAS scores, leg pain VAS scores, ODI scores, SVA, CVA, AVT, AVR, and coronal Cobb angles, but significantly lower JOA scores, SS, and LL values (all *P* < 0.05). No significant between-group differences were observed in age, sex, PI, PT, or TLK (all *P* > 0.05; Table 2).

**Table 1:** Baseline clinical and radiographic characteristics of patients with degenerative scoliosis (*n* = 163).

Variable	Value (X ±S)
Number	163
Age (year)	61.06 ± 8.44
Gender	107 F, 56 M
VAS score	
Back pain	7.67 ± 1.54
Leg pain	6.61 ± 1.61
Oswestry disability index, ODI (%)	73.89 ± 13.37
Japanese orthopedic association score, JOA score	4.80 ± 3.16
Sagittal vertical axis, SVA (cm)	4.37 ± 2.40
Coronal balance axis, CVA (cm)	2.59 ± 1.56
Apical vertebral translation, AVT (cm)	3.09 ± 1.37
Apical vertebral rotation, AVR (°)	16.66 ± 6.62
Pelvic incidence, PI (°)	51.40 ± 11.90
Pelvic tilt, PT (°)	24.05 ± 8.10
Sacral slope, SS (°)	26.96 ± 12.14
Lumbar lordosis, LL (°)	26.24 ± 15.03
Thoracolumbar kyphosis, TLK (°)	14.62 ± 10.27
Coronal cobb angle, cobb (°)	30.03 ± 8.85

ODI, oswestry disability index; JOA, Japanese orthopedic association; SVA, sagittal vertical axis; CVA, coronal balance axis; AVT, apical vertebral translation; AVR, apical vertebral rotation; PI, pelvic incidence; PT, pelvic tilt; SS, sacral slope; LL, lumbar lordosis; TLK, thoracolumbar kyphosis; Cobb, coronal cobb angle; Values are expressed as mean ± SD

**Table 2:** Comparison of clinical and radiographic characteristics between balanced and imbalanced groups.

Variable	Balanced group	Imbalanced group	*p* value
Number	72	91	-
Age (year)	60.04 ± 8.18	61.87 ± 8.60	0.171
Gender	45F, 27M	62F, 29M	-
VAS score			
Back pain	7.18 ± 1.50	8.07 ± 1.46	< 0.001
Leg pain	6.33 ± 1.08	6.82 ± 1.19	0.007
Oswestry disability index, ODI (%)	69.25 ± 13.33	77.55 ± 12.29	< 0.001
Japanese orthopedic association score, JOA score	6.25 ± 3.35	3.65 ± 2.46	< 0.001
Sagittal vertical axis, SVA (cm)	2.84 ± 1.08	5.58 ± 2.47	< 0.001
Coronal balance axis, CVA (cm)	1.69 ± 0.71	3.30 ± 1.68	< 0.001
Apical vertebral translation, AVT (cm)	2.79 ± 1.40	3.33 ± 1.31	0.012
Apical vertebral rotation, AVR (°)	13.96 ± 6.31	18.80 ± 6.08	< 0.001
Pelvic incidence, PI (°)	52.09 ± 11.96	50.85 ± 11.88	0.511
Pelvic tilt, PT (°)	22.73 ± 8.01	25.10 ± 8.02	0.064
Sacral slope, SS (°)	29.77 ± 11.28	24.74 ± 12.40	0.008
Lumbar lordosis, LL (°)	31.75 ± 15.69	21.89 ± 12.99	< 0.001
Thoracolumbar kyphosis, TLK (°)	15.50 ± 9.93	13.93 ± 10.54	0.335
Coronal cobb angle, cobb (°)	27.48 ± 9.07	32.04 ± 8.16	0.001

ODI, oswestry disability index; JOA, Japanese orthopedic association; SVA, sagittal vertical axis; CVA, coronal balance axis; AVT, apical vertebral translation; AVR, apical vertebral rotation; PI, pelvic incidence; PT, pelvic tilt; SS, sacral slope; LL, lumbar lordosis; TLK, thoracolumbar kyphosis; Cobb, coronal cobb angle; Values are expressed as mean ± SD; The comparison between the two groups was performed using an independent samples t-test.

### 3.2 Reproducibility of radiographic measurements

Thirty patients were randomly selected for repeated radiographic measurements. All radiographic parameters showed excellent reproducibility, with ICC(2,1) values ranging from 0.951 to 0.999. The ICC(2,1) for AVR was 0.999 (95% CI: 0.998–0.999), indicating excellent reproducibility of supine CT-measured AVR (Supplementary Table S1).

### 3.3 Correlation analysis of apical vertebral rotation

**Figure 3:**
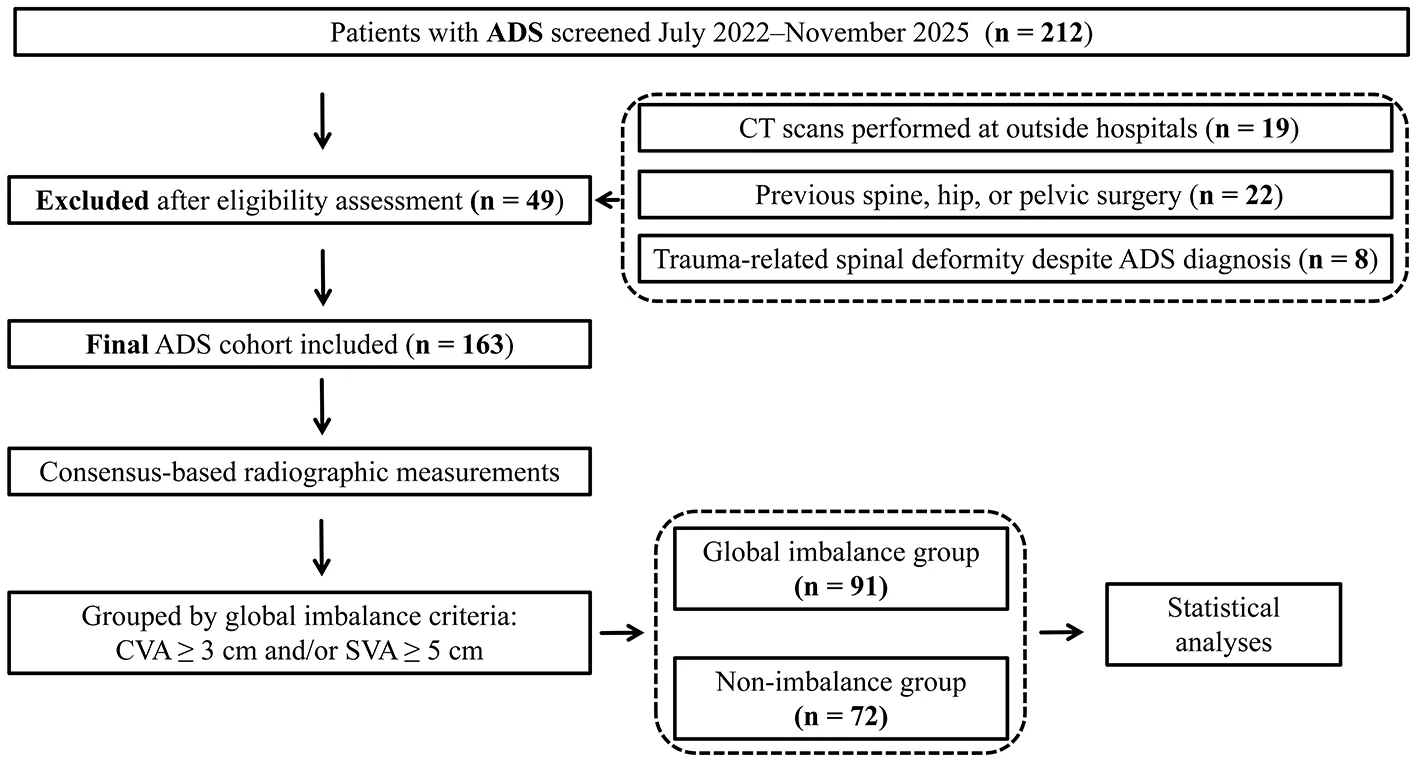
Correlation analysis.

Pearson correlation analysis was performed for continuous variables. AVR was correlated with several clinical and radiographic parameters. Among radiographic parameters, AVR was positively correlated with Cobb angle, AVT, CVA, and SVA, and negatively correlated with LL and TLK. Among clinical parameters, AVR was positively correlated with low back VAS, leg VAS, and ODI, and negatively correlated with JOA (Figure 3).

### 3.4 Radiographic factors independently associated with apical vertebral rotation

Based on the correlation results in Section 3.3, linear regression analysis was performed for radiographic parameters associated with AVR. Univariable analysis showed that SVA, CVA, AVT, LL, and Cobb angle were significantly associated with AVR (Table 3). These variables were then entered into a multivariable linear regression model. SVA, AVT, and Cobb angle remained independently associated with AVR, while CVA and LL were not statistically significant (Supplementary Table S2). All included variables had VIF values < 5, indicating no marked multicollinearity.

**Table 3:** Univariable linear regression analysis of radiographic factors associated with apical vertebral rotation.

Variable	*B*	95% CI	β	*p* value
Sagittal vertical axis, SVA (cm)	0.819	0.409–1.228	0.297	< 0.001
Coronal balance axis, CVA (cm)	0.899	0.255–1.543	0.212	0.007
Apical vertebral translation, AVT (cm)	2.31	1.651–2.969	0.479	< 0.001
Pelvic incidence, PI (°)	0.023	−0.064 to 0.11	0.041	0.601
Pelvic tilt, PT (°)	0.092	−0.034 to 0.218	0.113	0.153
Sacral slope, SS (°)	−0.06	−0.145 to 0.024	−0.11	0.161
Lumbar lordosis, LL (°)	−0.12	−0.186 to 0.054	−0.273	< 0.001
Thoracolumbar kyphosis, TLK (°)	−0.079	−0.178 to 0.021	−0.122	0.121
Coronal cobb angle, cobb (°)	0.352	0.249–0.455	0.47	< 0.001

SVA, sagittal vertical axis; CVA, coronal balance axis; AVT, apical vertebral translation; PI, pelvic incidence; PT, pelvic tilt; SS, sacral slope; LL, lumbar lordosis; TLK, thoracolumbar kyphosis; Cobb, coronal cobb angle.

### 3.5 Linear regression analysis with SVA and CVA as continuous dependent variables

In the univariable linear regression analysis with CVA as the dependent variable, SVA, AVR, LL, and Cobb angle were significantly associated with CVA (Table 4). In the multivariable analysis, only SVA remained independently associated with CVA. The independent association between AVR and CVA was not statistically significant (Supplementary Table S3). All included variables had VIF values < 5, indicating no marked multicollinearity.

**Table 4:** Univariable linear regression analysis with CVA as a continuous dependent variable.

Variable	*B*	95% CI	β	*p* value
Sagittal vertical axis, SVA (cm)	0.155	0.057–0.253	0.238	0.002
Apical vertebral rotation, AVR (°)	0.05	0.014–0.086	0.212	0.007
Apical vertebral translation, AVT (cm)	0.128	−0.048 to 0.304	0.113	0.153
Pelvic incidence, PI (°)	0.003	−0.018 to 0.023	0.022	0.781
Pelvic tilt, PT (°)	0.013	−0.017 to 0.043	0.068	0.386
Sacral slope, SS (°)	−0.008	−0.028 to 0.012	−0.062	0.431
Lumbar lordosis, LL (°)	−0.018	−0.034 to 0.002	−0.171	0.029
Thoracolumbar kyphosis, TLK (°)	−0.004	−0.027 to 0.02	−0.024	0.764
Coronal cobb angle, cobb (°)	0.035	0.009–0.062	0.201	0.01

SVA, sagittal vertical axis; AVR: apical vertebral rotation; AVT, apical vertebral translation; PI, pelvic incidence; PT, pelvic tilt; SS, sacral slope; LL, lumbar lordosis; TLK, thoracolumbar kyphosis; Cobb, coronal cobb angle.

In the univariable linear regression analysis with SVA as the dependent variable, CVA, AVR, LL, and Cobb angle were significantly associated with SVA (Table 5). In the multivariable analysis, CVA, AVR, and LL remained independently associated with SVA. AVR was independently and positively associated with SVA (Supplementary Table S4). All included variables had VIF values < 5, indicating no marked multicollinearity.

**Table 5:** Univariable linear regression analysis with SVA as a continuous dependent variable.

Variable	*B*	95% CI	β	*p* value
Coronal balance distance, CVA (cm)	0.367	0.134–0.599	0.238	0.002
Apical vertebral rotation, AVR (°)	0.108	0.054–0.162	0.297	< 0.001
Apical vertebral translation, AVT (cm)	0.225	−0.045 to 0.495	0.128	0.102
Pelvic incidence, PI (°)	0.013	−0.018 to 0.045	0.065	0.406
Pelvic tilt, PT (°)	0.022	−0.024 to 0.068	0.075	0.342
Sacral slope, SS (°)	−0.007	−0.038 to 0.024	−0.036	0.65
Lumbar lordosis, LL (°)	−0.044	−0.068 to 0.02	−0.273	< 0.001
Thoracolumbar kyphosis, TLK (°)	−0.019	−0.056 to 0.017	−0.083	0.293
Coronal cobb angle, cobb (°)	0.067	0.026–0.108	0.248	0.001

### 3.6 Logistic regression analysis of threshold-defined global imbalance

**Figure 4:**
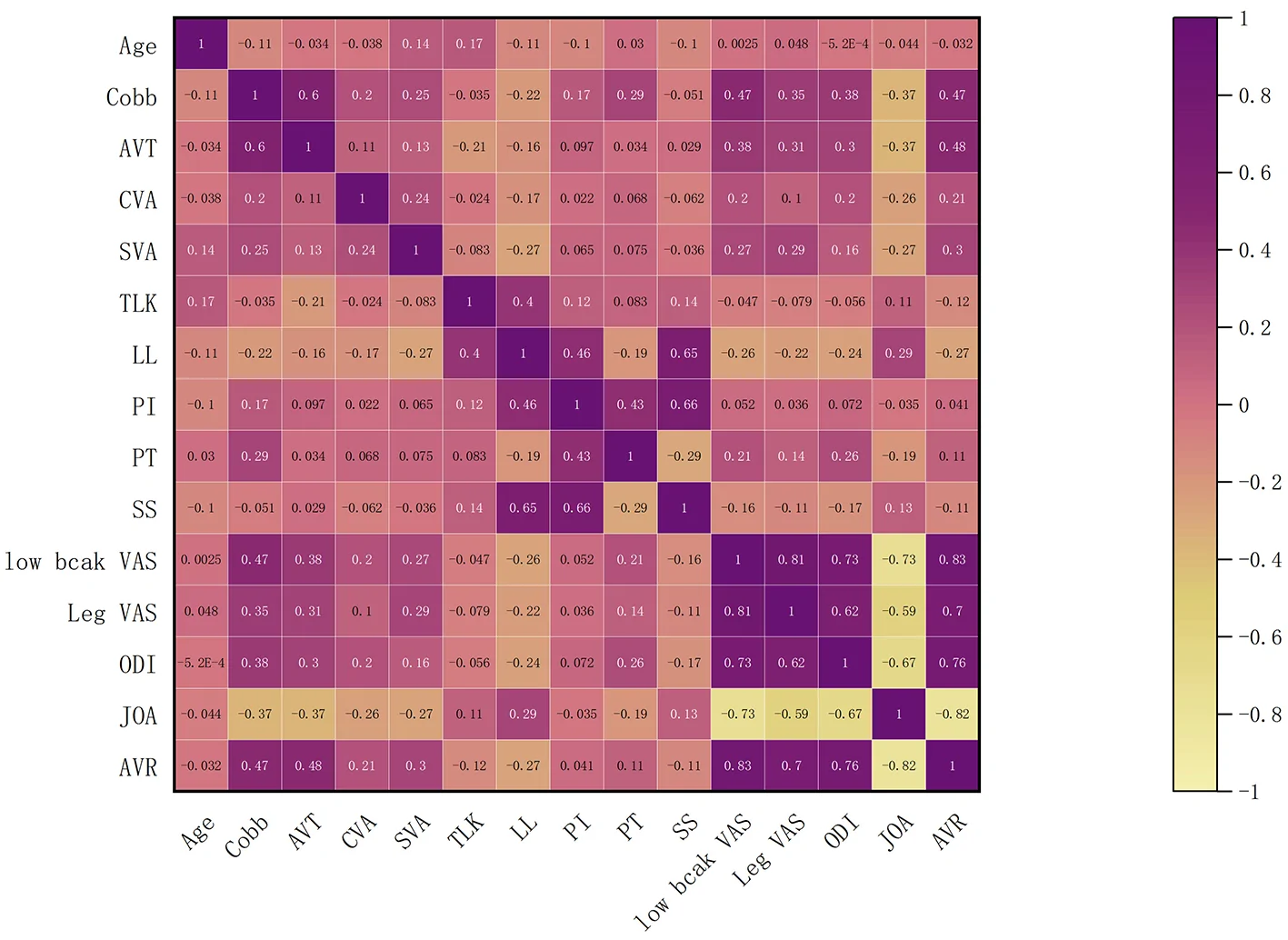
Forest plot of multivariate logistic regression.

Patients were classified into the global imbalance group and the non-imbalance group according to CVA ≥ 3 cm and/or SVA ≥ 5 cm. Univariable logistic regression showed that SVA, CVA, AVT, SS, LL, Cobb angle, and AVR were associated with global spinal imbalance (Table 6). Because SVA and CVA were used to define global imbalance, they were not included in the multivariable model. Multivariable logistic regression showed that AVR and LL were independently associated with global imbalance. AVR was independently and positively associated with global imbalance (OR = 1.108, 95% CI: 1.036–1.184, *P* = 0.003), while LL was independently and negatively associated with global imbalance (OR = 0.967, 95% CI: 0.935–0.999, *P* = 0.046; Figure 4 and Supplementary Table S5). The VIF values of variables included in the multivariable logistic regression model ranged from 1.448 to 1.928, indicating no marked multicollinearity.

**Table 6:** Univariable logistic regression analysis of threshold-defined global imbalance.

Variable	OR	95% CI	*p* value
Age (year)	1.026	0.989–1.065	0.171
Sagittal vertical axis, SVA (cm)	2.528	1.882–3.396	< 0.001
Coronal balance axis, CVA (cm)	3.034	2.082–4.420	< 0.001
Apical vertebral translation, AVT (cm)	1.359	1.064–1.736	0.014
Pelvic incidence, PI (°)	0.991	0.966–1.018	0.509
Pelvic tilt, PT (°)	1.039	0.997–1.082	0.067
Sacral slope, SS (°)	0.965	0.939–0.991	0.01
Lumbar lordosis, LL (°)	0.953	0.930–0.976	< 0.001
Thoracolumbar kyphosis, TLK (°)	0.985	0.956–1.015	0.334
Coronal cobb angle, cobb (°)	1.065	1.025–1.107	0.001
Apical vertebral rotation, AVR (°)	1.138	1.074–1.207	< 0.001

SVA, sagittal vertical axis; CVA, coronal balance axis; AVT, apical vertebral translation; AVR, apical vertebral rotation; PI, pelvic incidence; PT, pelvic tilt; SS, sacral slope; LL, lumbar lordosis; TLK, thoracolumbar kyphosis; Cobb, coronal cobb angle.

### 3.7 Sensitivity analysis

First, sagittal imbalance was defined using SVA ≥ 5 cm alone, and a multivariable logistic regression model was constructed in the full cohort (*N* = 163). After adjustment for Cobb angle, AVT, LL, and SS, AVR showed a positive trend toward association with sagittal imbalance, although this association did not reach statistical significance (OR = 1.058, 95% CI: 0.995–1.124, *P* = 0.072).

Second, to reduce the potential influence of cases with SVA or CVA values close to the clinical thresholds on the classification of global imbalance, logistic regression analysis was repeated after excluding these borderline cases. After exclusion, 88 patients remained in the analysis, including 37 patients with global imbalance and 51 patients without global imbalance. After adjustment for Cobb angle, AVT, LL, and SS, AVR remained associated with global spinal imbalance (OR = 1.094, 95% CI: 1.000–1.197, *P* = 0.049; Supplementary Table S6).

### 3.8 Exploratory interaction analysis

To explore possible interactions between AVR and global alignment parameters, multivariable logistic regression models were built with interaction terms. The interaction terms AVR × SVA and AVR × CVA were tested separately. The AVR × SVA interaction was not statistically significant (OR = 0.956, 95% CI: 0.901–1.015, *P* = 0.143). The AVR × CVA interaction was also not statistically significant (OR = 1.060, 95% CI: 0.939–1.196, *P* = 0.345). These results showed no significant interaction between AVR and SVA or CVA for global imbalance status. Because SVA and CVA were used to define global imbalance, this interaction analysis was exploratory and should be interpreted with caution (Supplementary Tables S7, S8).

## 4 Discussion

This study aimed to evaluate the clinical and radiographic significance of supine AVR in the three-dimensional assessment of ADS. The results showed that AVR was associated with low back pain, leg pain, functional disability scores, and several radiographic parameters. This suggests that AVR reflects not only local axial rotation but also clinical symptoms and abnormal global spinal alignment. Further analysis showed that SVA, AVT, and Cobb angle were independently associated with AVR. AVR remained independently associated with SVA in the continuous-variable analysis and was also independently associated with threshold-defined global imbalance. However, its independent association with CVA was not statistically significant. No significant interaction was found between AVR and SVA or CVA. These findings suggest that AVR is an important axial parameter in the three-dimensional assessment of ADS. However, it should not be used alone to explain global imbalance. It should be interpreted together with coronal and sagittal parameters and clinical symptoms.

### 4.1 Association of apical vertebral rotation with clinical manifestations and global imbalance in ADS

The results showed that apical vertebral rotation was associated with functional disability scores and several radiographic parameters in patients with ADS. This finding is consistent with previous studies (11, 14). These correlations may be partly explained by mechanical imbalance caused by the three-dimensional deformity in ADS. First, vertebral rotation may alter the mechanical balance of the paraspinal muscles and posterior ligament complex. This may lead to chronic overload of the muscles on one side and excessive tension of the interspinous and supraspinous ligaments, which can contribute to local myofascial inflammation and chronic axial pain (12, 15). Second, rotation compromises the stability of the posterior spinal elements, causing eccentric loading on the facet joints, accelerating cartilage degeneration and osteophyte formation, and potentially leading to subluxation and decreased segmental stability (5, 12). In addition, rotational deformity may expose the intervertebral disc to asymmetric compressive and shear forces. This may accelerate annular tears and nucleus pulposus degeneration (16), contributing to disc space narrowing, curve progression, and discogenic low back pain. The spinal canal has an inverted triangular shape, and vertebral rotation can cause localized narrowing. Facet joint hypertrophy and lateral vertebral translation (9) may further compress the lateral recess and neural foramen, leading to typical radicular symptoms such as leg pain and numbness (12).Therefore, vertebral rotation is associated not only with pain and functional status in ADS but also plays a meaningful role in disease progression and clinical decision-making. It should be carefully evaluated during diagnosis and treatment planning.

In the subsequent regression analyses, SVA, AVT, and Cobb angle were independently associated with AVR. This suggests that apical vertebral rotation reflects not only local axial rotation, but also sagittal alignment, coronal apical vertebral translation, and curve severity in ADS. Previous studies have identified SVA and CVA as important parameters for assessing global spinal alignment (8–10). The present findings suggest that AVR may provide additional information on axial deformity in the three-dimensional assessment of ADS. In the continuous-variable analysis, AVR was independently associated with SVA, but not with CVA. In the threshold-defined global imbalance analysis, AVR remained independently associated with imbalance status. No significant interaction was found between AVR and SVA or CVA. This suggests that the relationship between AVR and global imbalance may not be explained by a simple synergistic effect. In the pathological process of ADS, vertebral rotation is one manifestation of local three-dimensional deformity (9, 17). It may reflect the combined effects of asymmetric loading, segmental degeneration, and local structural remodeling. It may also further disturb the local mechanical environment. However, the effect of local rotational deformity on global spinal alignment may be influenced by several factors, including coronal curve severity, apical vertebral translation, lumbar lordosis, and compensatory mechanisms. Therefore, although AVR can reflect the severity of local deformity and is associated with global imbalance status, it should not be regarded as a single determinant of global imbalance. It should be noted that AVR was measured on supine CT in this study, whereas SVA, CVA, and other global alignment parameters were obtained from standing full-spine radiographs. Previous studies have shown that patients with adult spinal deformity may have marked differences in global spinal alignment between the supine and standing positions. This suggests that gravity loading and postural compensation can affect the assessment of spinal morphology (18). Another study comparing vertebral rotation between standing and supine positions found that the scoliotic curve and axial rotation may undergo partial spontaneous correction in the supine position, and that apical vertebral rotation measured in the standing position is greater than that measured in the supine position (19). Therefore, AVR measured on supine CT may underestimate the true axial rotation under standing weight-bearing conditions. When supine AVR is used in correlation or regression analyses with standing SVA and CVA, this postural difference may weaken the true association between AVR and global spinal alignment parameters and may affect its effect estimates in multivariable models. This factor may influence the interpretation of the independent association between AVR and CVA and the interaction results between AVR and SVA/CVA in the present study. Meanwhile, several potential clinical confounders not included in the models should be considered. Previous studies have suggested that paraspinal muscle degeneration may be associated with coronal imbalance and altered spinal stability in patients with adult degenerative scoliosis (20); reduced bone mineral density or osteoporosis may also affect sagittal alignment and mechanical stability in adult spinal deformity (21); and pelvic and lower-limb compensatory mechanisms, such as pelvic retroversion and hip/knee flexion, may contribute to the maintenance of standing global balance (22). In addition, BMI may influence the achievement of alignment targets and compensatory status in patients with adult spinal deformity, suggesting that body habitus may also have a potential impact on global alignment assessment (23). Therefore, these factors may, to some extent, influence the effect estimates for the associations between AVR and SVA, CVA, or global imbalance. Because this was a retrospective study, these data were not completely available and therefore could not be included in the multivariable adjusted models. As a complementary analysis, we performed a sensitivity analysis by excluding patients with values near the clinical thresholds to reduce the potential influence of classification instability on the main findings. The results showed that AVR remained associated with global imbalance, suggesting a certain degree of robustness of the main findings; however, this analysis cannot replace direct adjustment for clinical confounders. Therefore, our findings should be interpreted as the association between supine CT-measured AVR and standing global spinal alignment, rather than as the true mechanical relationship between axial rotation and global imbalance under weight-bearing conditions. Overall, the correlation analyses, multivariable regression models, and interaction analyses in this study suggest that AVR has value as a complementary parameter in the three-dimensional assessment of ADS. Its clinical significance should be interpreted together with coronal and sagittal parameters and patient symptoms.

### 4.2 Clinical relevance of apical vertebral rotation in the management and surgical planning of ADS

Based on the present findings, assessment of axial rotation should be considered in the management of ADS. Although AVR, Cobb angle, AVT, SVA, and CVA describe interrelated components of the same three-dimensional deformity, they quantify different anatomical planes and are not interchangeable. Cobb angle and AVT primarily characterize coronal deformity, whereas SVA and CVA reflect global sagittal and coronal alignment, respectively. In contrast, AVR directly quantifies the axial component of the deformity, which has received comparatively less attention in previous studies. The observed associations between AVR and low back pain, leg pain, ODI, and JOA further suggest that axial rotation is not merely a radiographic finding but is clinically related to patient symptoms and functional impairment. During conservative treatment or follow-up, AVR may serve as a complementary parameter for deformity assessment. In patients with little change in Cobb angle or inconsistent radiographic findings, AVR assessment may help identify axial rotational deformity and local structural abnormalities. It may also provide information beyond coronal plane measurements alone and reduce the risk of overlooking clinically relevant deformity when the Cobb angle appears stable. However, because this was a cross-sectional study, we cannot confirm that changes in AVR predict deformity progression. In surgical treatment, preoperative assessment of AVR may provide information for pedicle screw trajectory planning, decompression planning, and derotation strategy. Vertebral rotation may also create technical challenges. For example, it can alter the pedicle screw trajectory and increase the difficulty and risk of screw placement (24). It also complicates decompression of the nerve root canal and lateral recess, with a higher likelihood of residual compression (25). Additionally, rotation affects rod placement and may limit derotation effectiveness, contributing to postoperative loss of correction (26).To address these challenges, several derotation techniques have been developed, including direct vertebral derotation (27), bilateral apical vertebral derotation (28), and vertebral coplanar alignment techniques (29). All aim to address the complexities of three-dimensional deformity correction. Based on both our findings and clinical experience, successful derotation depends on adequately addressing the structural remodeling associated with vertebral rotation (7). Before applying these corrective techniques, it is essential to release contracted posterior soft tissues and ligaments, remove hypertrophic facet joints, and fully mobilize the intervertebral space—including resection of concave annular fibrosis and osteophytes. These steps restore spinal flexibility and reduce resistance to derotation (7).Our previously published work demonstrated that adequate release of these asymmetric and rigid structures can create sufficient space for deformity correction, reduce the need for higher-grade osteotomy, and allow satisfactory correction to be achieved using asymmetric or lower-grade osteotomy techniques (7). Accordingly, assessment of AVR and its associated local structural changes may provide clinically relevant information for determining the extent of release, selecting the osteotomy strategy, planning pedicle screw trajectories and neural decompression, and designing the overall three-dimensional correction. Overall, the present findings support AVR as a clinically relevant complementary component of ADS assessment and operative planning, rather than as a replacement for SVA, CVA, AVT, or Cobb angle. Its clinical value is not limited to its correlations with conventional radiographic parameters. AVR directly characterizes the axial component of the deformity, which is associated with patient symptoms and functional impairment and is encountered and actively addressed during surgical correction.

### 4.3 Limitations and future directions

This study has several limitations. First, it was a single-center, retrospective, cross-sectional study. Therefore, the observed relationships between AVR, clinical symptoms, and radiographic parameters should be interpreted as associations rather than causal effects. The cross-sectional design also precluded assessment of whether AVR predicts subsequent deformity progression. Second, several potential confounding factors were not included, such as paraspinal muscle degeneration, bone mineral density, and pelvic or lower-limb compensation. These factors may affect global alignment and compensatory capacity in patients with ADS (30). Third, AVR was measured on supine CT, whereas SVA, CVA, and other global alignment parameters were obtained from standing full-spine radiographs. This discrepancy between non-weight-bearing and weight-bearing conditions may lead to underestimation of axial vertebral rotation and may affect the estimated associations between AVR and standing global alignment. Standing low-dose biplanar stereoradiography, such as EOS imaging, may help address this limitation by allowing axial, coronal, and sagittal parameters to be evaluated simultaneously under weight-bearing conditions. However, EOS-based AVR measurements require further validation against CT in patients with ADS, because osteophyte formation, vertebral remodeling, endplate irregularity, and other degenerative changes may affect anatomical landmark identification and three-dimensional reconstruction accuracy. Finally, this study assessed only apical vertebral rotation. It did not analyze the segmental distribution of vertebral rotation within the scoliotic curve. Therefore, AVR may not fully represent the overall axial rotational deformity in ADS (29, 31). Future prospective, multicenter, and longitudinal studies should use validated weight-bearing three-dimensional imaging to evaluate AVR and global spinal alignment under the same biomechanical conditions. Further investigation of segmental vertebral rotation (31), biomechanical mechanisms, and longitudinal clinical outcomes may help clarify the role of axial deformity in ADS progression and improve individualized treatment planning (32, 33). Future studies should also evaluate whether AVR-based surgical planning provides incremental benefits in treatment selection, screw-placement accuracy, decompression adequacy, osteotomy selection, three-dimensional correction, complication reduction, and patient-reported outcomes.

## 5 Conclusion

This study showed that supine CT-measured AVR was associated with clinical symptoms, functional disability scores, and several radiographic parameters in patients with ADS. AVR was independently associated with SVA, AVT, and Cobb angle. It also remained associated with threshold-defined global imbalance. Sensitivity analyses suggested a certain degree of robustness, and reproducibility testing demonstrated excellent reliability for AVR and other key radiographic measurements. However, its independent association with CVA was not statistically significant, and no significant interaction was found between AVR and SVA or CVA. These findings suggest that AVR is an important axial parameter in the three-dimensional assessment of ADS. It may provide additional information beyond coronal and sagittal assessment and offer radiographic reference information for disease management in patients with ADS. However, because AVR was measured on supine CT whereas SVA/CVA were obtained from standing full-spine radiographs, and because some clinical confounders were unavailable for adjustment, the findings should be interpreted as associations between supine AVR and standing global spinal alignment rather than as causal evidence. Therefore, AVR should not be regarded as a single determinant of global imbalance or an independent predictor of deformity progression. Future prospective multicenter studies using validated weight-bearing three-dimensional imaging are needed to assess AVR and global spinal alignment under the same biomechanical conditions.

## Data Availability

The raw data supporting the conclusions of this article will be made available by the authors, without undue reservation.
