# Supplementary material for: Association of supine apical vertebral rotation with three-dimensional deformity and global spinal alignment in adult degenerative scoliosis: a retrospective study
Source: Front Med (Lausanne). 2026 Aug 19;13:1919114. doi: 10.3389/fmed.2026.1919114 (PMC13534026; doi:10.3389/fmed.2026.1919114)
Supplement: Supplementary file 1 [file Supplementary_file_1.docx]

**Table S1** Reproducibility of radiographic measurements based on consensus assessment

| **Parameter** | **ICC(2,1)** | **95% CI** |
| --- | --- | --- |
| Cobb angle | 0.991 | 0.980–0.995 |
| AVT | 0.993 | 0.984–0.996 |
| CVA | 0.953 | 0.904–0.977 |
| SVA | 0.990 | 0.979–0.995 |
| TLK | 0.960 | 0.918–0.981 |
| LL | 0.951 | 0.900–0.976 |
| PI | 0.967 | 0.932–0.984 |
| PT | 0.971 | 0.940–0.986 |
| SS | 0.973 | 0.944–0.987 |
| AVR | 0.999 | 0.998–0.999 |

***Note:*** ICC(2,1) was calculated using a two-way random-effects model with absolute agreement for single measurements. **ICC:** intraclass correlation coefficient; **CI:**confidence interval; **AVT:**apical vertebral translation; **CVA:** coronal vertical axis; **SVA:** sagittal vertical axis; **TLK:** thoracolumbar kyphosis; **LL:** lumbar lordosis; **PI:** pelvic incidence; **PT:** pelvic tilt; **SS:** sacral slope; **AVR:** apical vertebral rotation.

**Table S2** Multivariable Linear Regression Analysis of Radiographic Factors Independently Associated With Apical Vertebral Rotation

| **Variable** | | **B** | **S.E.** | **95%CI** | **β** | **p Value** |
| --- | --- | --- | --- | --- | --- | --- |
| **Sagittal Vertical Axis, SVA (cm)** | 0.425 | | 0.193 | 0.045–0.806 | 0.154 | 0.029 |
| **Coronal Balance Axis, CVA (cm)** | 0.332 | | 0.287 | -0.235–0.900 | 0.078 | 0.249 |
| **Apical Vertebral Translation, AVT (cm)** | 1.485 | | 0.39 | 0.714–2.256 | 0.308 | <0.001 |
| **Lumbar Lordosis, LL (°)** | -0.054 | | 0.03 | -0.114–0.006 | -0.123 | 0.075 |
| **Coronal Cobb Angle,Cobb ( °)** | 0.154 | | 0.063 | 0.030–0.278 | 0.205 | 0.015 |

VIF = 1.094–1.668

***Note:* SVA:**Sagittal Vertical Axis; **CVA:**Coronal Balance Axis; **AVT:**Apical Vertebral Translation; **LL**:Lumbar Lordosis; **Cobb:**Coronal Cobb Angle

**Table S3** Multivariable Linear Regression Analysis With CVA as a Continuous Dependent Variable

| **Variable** | **B** | **S.E.** | **95%CI** | **β** | **p Value** |
| --- | --- | --- | --- | --- | --- |
| **Sagittal Vertical Axis, SVA (cm)** | 0.107 | 0.053 | 0.002-0.211 | 0.164 | 0.046 |
| **Apical Vertebral Rotation, AVR (°)** | 0.023 | 0.021 | -0.019-0.064 | 0.096 | 0.282 |
| **Lumbar Lordosis, LL (°)** | -0.008 | 0.008 | -0.025-0.008 | -0.079 | 0.33 |
| **Coronal Cobb Angle,Cobb ( °)** | 0.017 | 0.015 | -0.013-0.048 | 0.098 | 0.261 |

VIF = 1.137–1.377

***Note:* SVA:**Sagittal Vertical Axis;**AVR:**Apical Vertebral Rotation; **LL**:Lumbar Lordosis; **Cobb:**Coronal Cobb Angle

**Table S4** Multivariable Linear Regression Analysis With SVA as a Continuous Dependent Variable

| **Variable** | **B** | **S.E.** | **95%CI** | **β** | **p Value** |
| --- | --- | --- | --- | --- | --- |
| **Coronal Balance Distance, CVA (cm)** | 0.233 | 0.116 | 0.004-0.463 | 0.152 | 0.046 |
| **Apical Vertebral Rotation, AVR (°)** | 0.062 | 0.031 | 0.001-0.122 | 0.169 | 0.047 |
| **Lumbar Lordosis, LL (°)** | -0.029 | 0.012 | -0.053--0.005 | -0.18 | 0.02 |
| **Coronal Cobb Angle,Cobb ( °)** | 0.027 | 0.023 | -0.018-0.072 | 0.099 | 0.237 |

VIF = 1.075–1.353

***Note:* CVA:**Coronal Balance Axis;**AVR:**Apical Vertebral Rotation; **LL**:Lumbar Lordosis; **Cobb:**Coronal Cobb Angle

**Table S5** Multivariable Logistic Regression Analysis of Threshold-Defined Global Imbalance

| **Variable** | **B** | **S.E.** | **p Value** | **OR** | **95%CI** |
| --- | --- | --- | --- | --- | --- |
| **Coronal Cobb Angle,Cobb ( °)** | 0.025 | 0.026 | 0.343 | 1.025 | 0.974–1.079 |
| **Apical Vertebral Translation, AVT (cm)** | -0.023 | 0.175 | 0.896 | 0.977 | 0.693–1.378 |
| **Lumbar Lordosis, LL (°)** | -0.034 | 0.017 | 0.046 | 0.967 | 0.935–0.999 |
| **Sacral Slope, SS (°)** | -0.007 | 0.021 | 0.736 | 0.993 | 0.953–1.035 |
| **Apical Vertebral Rotation, AVR (°)** | 0.102 | 0.034 | 0.003 | 1.108 | 1.036–1.184 |

VIF=1.448–1.928

***Note:* AVT:**Apical Vertebral Translation; **AVR:**Apical Vertebral Rotation; **LL**:Lumbar Lordosis; **Cobb:**Coronal Cobb Angle；**SS**:Sacral Slope;

**Table S6 Sensitivity analyses of the association between AVR and spinal imbalance**

| **Sensitivity analysis** | **Study population** | **Outcome definition** | **Imbalance / non-imbalance, n** | **Adjusted covariates** | **OR** | **95% CI** | **p Value** |
| --- | --- | --- | --- | --- | --- | --- | --- |
| Analysis 1 | Full cohort | Sagittal imbalance, defined as SVA ≥ 5 cm | 61 / 102 | Cobb angle, AVT, LL, SS | 1.058 | 0.995–1.124 | 0.072 |
| Analysis 2 | After excluding borderline cases near clinical thresholds | Global imbalance, defined as CVA ≥ 3 cm and/or SVA ≥ 5 cm | 37 / 51 | Cobb angle, AVT, LL, SS | 1.094 | 1.000–1.197 | 0.049 |

***Note:* AVR:**apical vertebral rotation**; SVA:**sagittal vertical axis**; CVA:**coronal vertical axis**; AVT:** apical vertebral translation**; LL:** lumbar lordosis**; SS:** sacral slope**; OR:** odds ratio**; CI:** confidence interval.

**Table S7**  Multivariate logistic regression analysis of the interaction between sagittal vertical axis and vertebral rotation.

| **Variable** | **OR** | **95%CI** | ***p* Value** |
| --- | --- | --- | --- |
| **Main Effects** |  |  |  |
| **Coronal Cobb Angle,Cobb ( °)** | 1.003 | 0.93-1.081 | 0.936 |
| **Apical Vertebral Translation, AVT (cm)** | 1.534 | 0.909-2.588 | 0.109 |
| **Coronal Balance Axis, CVA (cm)** | 11.879 | 4.115-34.291 | ＜0.001 |
| **Lumbar Lordosis, LL (°)** | 0.967 | 0.914-1.022 | 0.234 |
| **Sacral Slope, SS (°)** | 0.974 | 0.902-1.053 | 0.512 |
| **Apical Vertebral Rotation, AVR (°)** | 0.967 | 0.871-1.074 | 0.535 |
| **Sagittal Vertical Axis, SVA (cm)** | 5.138 | 2.612-10.106 | ＜0.001 |
| **Interaction Effect** |  |  |  |
| **AVR × SVA Interaction** | 0.956 | 0.901-1.015 | 0.143 |

***Note:* SVA:**Sagittal Vertical Axis; **CVA:**Coronal Balance Axis; **AVT:**Apical Vertebral Translation; **AVR:**Apical Vertebral Rotation; **PI:**Pelvic Incidence; **PT** :Pelvic Tilt;**SS**:Sacral Slope; **LL**:Lumbar Lordosis; **TLK:**Thoracolumbar Kyphosis; **Cobb:**Coronal Cobb Angle

**Table S8**  Multivariate logistic regression analysis of the interaction between coronal balance axis and vertebral rotation.

| **Variable** | **OR** | **95%CI** | ***p* Value** |
| --- | --- | --- | --- |
| **Main Effects** |  |  |  |
| **Coronal Cobb Angle,Cobb ( °)** | 1.004 | 0.931-1.083 | 0.914 |
| **Apical Vertebral Translation, AVT (cm)** | 1.515 | 0.892-2.571 | 0.124 |
| **Coronal Balance Axis, CVA (cm)** | 12.501 | 4.046-38.63 | ＜0.001 |
| **Lumbar Lordosis, LL (°)** | 0.967 | 0.915-1.023 | 0.246 |
| **Sacral Slope, SS (°)** | 0.978 | 0.905-1.056 | 0.569 |
| **Apical Vertebral Rotation, AVR (°)** | 1.014 | 0.901-1.141 | 0.816 |
| **Sagittal Vertical Axis, SVA (cm)** | 5.023 | 2.572-9.808 | ＜0.001 |
| **Interaction Effect** |  |  |  |
| **AVR × CVA Interaction** | 1.06 | 0.939-1.196 | 0.345 |

***Note:* SVA:**Sagittal Vertical Axis; **CVA:**Coronal Balance Axis; **AVT:**Apical Vertebral Translation; **AVR:**Apical Vertebral Rotation; **PI:**Pelvic Incidence; **PT** :Pelvic Tilt;**SS**:Sacral Slope; **LL**:Lumbar Lordosis; **TLK:**Thoracolumbar Kyphosis; **Cobb:**Coronal Cobb Angle;
